# The Forthcoming State Register

**Published:** 1921-06-11

**Authors:** 


### THE MATRONS' AND SISTERS' DEPARTMENT.

### The Forthcoming State Register.

The State Register of Nurses will very shortly open its lists for such as are qualified to enter their names. Its success will undoubtedly depend upon the extent to which trained nurses avail themselves of the privilege which has been won for them. And that again would seem to depend upon the attitude which members of the College of Nursing adopt towards the new Register.

The founding of the College of Nursing awoke in the nursing world an immense wave of enthusiasm for the principle of State Registration. Nurses rallied to the College in the hope of speedily obtaining this boon from the Government, and their hopes were not disappointed. A movement which had languished for some thirty years without enlisting any widespread support became instantly popular directly the College was started, and after three years of its existence became crowned with success. The inevitable reaction set in almost at once, and it is possible to discern already a great weakening of interest in a boon not even yet within grasp.

It is not a British characteristic to agitate violently for a measure and then neglect to take advantage of its provisions. When members of the College express indifference to the proceedings of the General Nursing Council and ask themselves what good it can do them to be included among a list of registered persons, many of whom have far lower qualifications than themselves, they forget altogether that State Registration, with all its inevitable shortcomings, is, after all, the result of their own petitions. There were not wanting many warnings at the time when it was granted, many forecasts that "existing nurses," however poorly equipped for their duties according to modern notions, must yet be allowed to place their names on the State Register. It is too late in the day to flout the decisions of the Crown lawyers and reject the provisions sanctioned by the nurses' own delegates in the General Nursing Council. It is the plain duty of all nurses who have the unity of the profession at heart to support the State Register with the same loyalty they have accorded to their own College. It is more clear than it ever was that State Registration can never take the place of the College of Nursing or perform those valuable services for the nursing profession which the College is now carrying out. But it is undeniable that State Registration as it will shortly be offered to British nurses is the outcome of the movement which started the College. To go back upon it now is to throw over the entire nursing profession the stigma of instability and lack of good faith.

